# Aligning an undergraduate psychological medicine subject with the mental health needs of the local region

**DOI:** 10.1186/s12909-018-1192-4

**Published:** 2018-05-31

**Authors:** Christopher Rikard-Bell, Torres Woolley

**Affiliations:** College of Medicine and Dentistry, James Cook University, QLD, Townsville, 4811 Australia

**Keywords:** Psychological, Medicine, Undergraduate, Student, Rural, Mental, Health, Curriculum

## Abstract

**Background:**

The James Cook University (JCU) medical school recently revised its Year 2 human development and behaviour module to be more relevant and practical for students, and more aligned with the mental health priorities of the local region (north Queensland). This study reports medical students’ level of preparedness conferred by the re-designed ‘Psychological Medicine and Human Development’ (PMHD) subject for their later 4-week, rural clinical placement in Year 2.

**Methods:**

Non-randomized, controlled ‘naturalistic’ study with pre- and post-intervention surveys. The patient mental health experiences of Year 2 students who went on clinical placement after undertaking the PMHD subject were compared to those who went on placement before undertaking PMHD.

**Results:**

A total of 209 JCU Year 2 medical students completed surveys from a possible 217 (response rate = 96%). Compared to students whom had not taken PMHD before going on placement, students going on placement after undertaking PMHD were significantly more likely to report: feeling comfortable discussing mental health issues with patients (*p* = 0.001); being prepared for mental health discussions with patients (*p* < 0.001); having an actual mental health discussion with a patient (*p* < 0.001); and, volunteering an opinion on the appropriateness of their supervising doctor’s response (*p* < 0.001). Students reported subject content involving information and classroom instruction on assessing and interviewing patients for mental illness to be of most use.

**Conclusions:**

Providing medical students with psychological medicine information on locally prevalent mental health conditions plus practical classroom experiences in conducting mental state exams better prepares them for interacting with patients experiencing psychological distress. This novel methodology – aligning formal teaching in a subject with an evaluation utilizing a proximate student placement to provide useful feedback on the curriculum content and assess the relevance of the material taught – could be used to revise other content areas of a medical course to be more locally relevant and practically focused, and then to evaluate the success of this revision.

## Background

Ensuring medical education reflects and is responsive to the priority healthcare needs of local communities is becoming a focus for reform internationally. However, many medical schools, charged with producing tomorrow’s doctors, have been criticised for having a curriculum that does not always reflect the priority needs of the local region, or give priority to the most vulnerable individuals and groups in society [1].

However, the undergraduate entry Bachelor of Medicine, Bachelor of Surgery (MBBS) James Cook University (JCU) medical school was established in North Queensland (NQ) in 2000 [2] with a curriculum aligned with the health priorities of the north Queensland/northern Australian region, in particular, Aboriginal and Torres Strait Islander peoples – the most disadvantaged and vulnerable group in the country.

Due to this ‘socially-accountable’ philosophy, in 2008 the JCU medical school joined a world-wide consortium of 12 health professional schools called the Training for Health Equity Network (THEnet). THEnet is committed to achieving health equity through transforming health professional education to meet local needs [3, 4]. All 12 THEnet medical schools have similar socially-accountable philosophies and curriculum approaches, including equitable selection processes for admitting rural applicants and/or applicants from underserved sub-populations, a curriculum that is responsive to the priority health issues of the local region, and mandatory service learning for students in rural communities involving significant involvement with local community members.

For example, the JCU medical curriculum includes the requirement that all Year 2 students must pass the ‘Rural, Remote, Indigenous and Tropical Health’ subject, as well as a mandatory 4-week rural placement to introduce them to local rural communities and their health issues (including 1 week in an Aboriginal Medical Service), and have experiences in rural hospital wards and rural medical centres focussed on patient history-taking and examinations, including around mental health.

Also in Year 2, the JCU medical school delivers a psychological medicine subject, which is part of an overall linkage of psychological issues that is built upon by later modules and curriculum activities in the 6 year course. Having a subject dedicated to psychological medicine at the JCU medical school is important. Not only is the base rate of mental health disorders in the Australian community high, with a lifetime risk of 45% for adults aged 16–85 [5], but regional and remote areas of Australia are at even greater risk for these disorders [6]. In addition, it is well recognised that Aboriginal and Torres Strait Islander people in remote and regional areas have alarmingly high levels of psychiatric disorders. Aboriginal and Torres Strait Islander people are particularly vulnerable to experiencing high levels of stress [7] and serious mental illness such as psychosis, with a rate of almost 15 times the non-Indigenous population [8] (44.9 per 1000 population compared to 2.9 per 1000 population for non-Indigenous young people Australia wide).

JCU’s psychological medicine subject was re-designed in 2013 from one with a predominant focus on human development and behaviour into the current ‘Psychological Medicine and Human Development’ (PMHD) subject. This revised PMHD subject predominantly teaches students ‘biopsychosocial’ perspectives of health (the psychological and behavioural factors influencing health and illness), as well as the more general, practical skills required to interview and diagnose patients with a mental health condition. This re-design was undertaken in part to better align the subject with the medical school’s ‘socially-accountable’ mandate of addressing the priority health needs of the local region and the most vulnerable Aboriginal and Torres Strait Islander populations. In addition, the re-design was undertaken to make the subject more practical, as there is evidence that developmental and mental health subjects in early medical courses are commonly perceived by students to be of little relevance or value [9], even though studies demonstrate high levels of mental health disorders treated by doctors in hospitals [10] and in the community (particularly in rural general practice) [11].

This re-design was informed by student feedback sessions immediately after their Year 2 rural community placements in 2013, which discussed the frequency and variety of patient mental health presentations seen on placement, and the competencies required to undertake a focused mental health history and examination. On Year 2 community placements, students are required to undertake history and examinations on the rural general practice patient population, which is known to include a high proportion of patients experiencing psychological distress, as well as a high proportion of Aboriginal and Torres Strait Islander peoples whom many also experience psychological distress. For example, in the remote region of Mount Isa, where about 25% of JCU Year 2 medical students spend their Year 2 community placement, 23% of the population are Aboriginal and Torres Strait Islander peoples; much higher than the national average of 3% (http://www.censusdata.abs.gov.au/census_services/getproduct/census/2016/quickstat/SED30057). Discussions highlighted the high incidence of students coming into contact with patients experiencing psychological distress, and students’ lack of confidence and practical experience in using standard techniques for assessing patients’ current mental or cognitive state. As a result of these discussions, the PMHD subject was revised with a greater focus on depression, anxiety and suicidal thoughts, plus teaching students general skills in mental health assessment; for example, practical techniques for interviewing and assessing patients with disorders of mental health (techniques for assigning diagnoses appropriately were a secondary consideration). Thus, student discussions directly resulted in the PMHD subject being re-designed to include content not just more relevant to the mental health needs of the local region, but practical enough so that JCU medical students can practice and further develop patient-centred competencies associated with mental health while on rural and remote community placements.

While all Year 2 JCU medical students undertake patient history-taking and examinations during their mandatory 4-week placement in a rural community, only half the student cohort experiences the PMHD subject before their Year 2 placement. This allowed the present study to approximate the level of preparedness conferred to JCU medical students from the PMHD subject in regards to mental health interviewing, history-taking and examinations.

## Methods

### Study design

This 2014–15 study of JCU MBBS students utilized a non-randomized controlled design to compare the patient mental health experiences of 91 Year 2 students who went on rural placement before undertaking the PMHD subject, to 118 Year 2 students who went on rural placement after undertaking PMHD. This design was possible due to the fortuitous timing of the compulsory 4-week rural placement in the broader Year 2 medical curriculum. Half the student cohort completed their rural placement before the psychological medicine subject, and the other half completed the placement after due to logistical reasons. This serendipitously allowed the opportunity of a ‘natural’ experiment to evaluate the efficacy of the PMHD subject involving two equivalent groups of students. Ethical approval for the study was obtained from the James Cook University Human Ethics Committee (case number H3031).

### Description of the PMHD subject

The aim of the PMHD course is to educate students around psychological medicine being an essential cornerstone of medical practice. Arguably, all medical interactions between patients and doctors involve the patient’s understanding and psychological reaction to illness. Thus, psychological medicine could be considered the tapestry upon which clinical medicine exists. The more medical students are introduced to psychological medicine in a practical and relevant way early in their undergraduate course, the more it will be understood and integrated into their future patient encounters. As students progress through their medical training and experience increasingly complex patient encounters, it is expected they will appreciate more and more the integral nature of psychological medicine in medical practice.

PMHD approaches psychological medicine from a developmental perspective; which is in keeping with the previous human development and behaviour course, as development is the core theme of the subject. However, the subject was modified into PMHD by further incorporating relevant and practical medical perspectives for students to better appreciate the value of psychological medicine in all settings, particularly rural and remote medicine.

PMHD draws together the biological developmental underpinnings with psychological development to promote the view of individual patients as a ‘bio-psycho-social’ entity. PMHD emphasises the psychological disturbances at each stage of development, and its relation to developing psychological disorder at different ages. There is exploration of how psychological health, illness and behaviour are closely related. Students are led to the logical conclusion that an understanding of psychological medicine, in combination with a strong therapeutic relationship between the doctor and the patient, is critical to achieving good patient outcomes.

In addition to the theoretical underpinnings that explain how biological and psychological developmental problems can contribute to illness, there is also an emphasis on developing practical skills in mental state examinations (MSE), cognitive assessments, and learning practical formulation methods to summarise each psychological disturbance. Lastly, the PMHD course highlights the importance of caring for one’s own psychological health in order to be able to function well as a doctor and student.

### Participants and data collection

All Year 2 medical students at JCU in 2014 were invited to complete a survey during their post-rural placement de-brief session held several weeks after returning from their placement. About half the Year 2 JCU medical student cohort undertake their 4-week rural placement in June (before receiving the PMHD subject from July to October that year), while the other half do their Year 2 placement in December or January (after receiving the PMHD subject). Students were informed of the project aims, and that completion of the survey was voluntary.

### Survey

As there are currently no validated surveys in the literature to evaluate how well health students undertake a mental health history-taking and examination of patients, the survey used in the present study was developed from a focus group discussion with 5 Year 3 students in the year previous (2013) to the data collection phase. The focus group discussions determined exactly how Year 3 students felt the recently re-designed PMHD subject had benefitted them on their rural clinical placement they undertook at the end of their Year 2, and how the PMHD course could be improved. The discussion led to improvements being made to the PMHD subject to incorporate more content and competencies around interviewing mental health patients, and around depression and anxiety. The discussion also led to the development of the content and wording for the evaluation tool. The evaluation tool was then trialled on the Year 2 students later that year, and several adjustments made to the wording of the survey questions for greater clarity.

In mid-2014, the evaluation tool was initially given to students who did not receive the PMHD subject prior to their rural placement. The evaluation tool included 3 four-point Likert scale questions, 8 ‘yes/no’ answer questions, and an open-ended question asking “Why did/didn’t you feel prepared for discussions with patients around mental health issues?” (see Table 1 for a complete list of all survey questions and wording). The evaluation tool was also given at the beginning of 2015 to students who received the PMHD subject prior to their rural placement at the end of 2014. This evaluation tool also included a further 2 open-ended questions: “Was any information provided in the PMHD subject relevant to patient discussions around mental health on your Year 2 placement?”, and “Did any of the practical skills provided by the PMHD subject come in useful on your Year 2 placement?”

**Table 1:** Year 2 rural clinical placement experiences of JCU MBBS student with patients experiencing mental health issues, PRE and POST the Year 2 Psychological Medicine and Human Development (PMHD) subject

Question	Completed Year 2 rural clinical placement PRE PMHD subject (*n* = 91)	Completed Year 2 rural clinical placement POST PMHD subject (*n* = 118)	*p*-value^a^
How confident were you that you could detect patients with a Mental Health problem? (1 = ‘not at all’, 2 = ‘a little’, 3 = ‘quite’, 4 = ‘very’)	2.2	2.8	< 0.001
How comfortable were you discussing Mental Health issues with a patient? (1 = ‘not at all’, 2 = ‘a little’, 3 = ‘quite’, 4 = ‘very’)	2.5	2.9	0.001
How well prepared were you for Mental Health discussions with patients? (1 = ‘not at all’, 2 = ‘a little’, 3 = ‘quite’, 4 = ‘very’)	1.8	2.6	< 0.001
Did you sit in with a General Practitioner during consults?	Yes – 79%	Yes – 86%	0.219
Was the topic of Mental Health raised during the consult?	Yes – 92%	Yes – 94%	0.541
Did you feel the General Practitioner’s responses in the patient consult appropriate?	‘Don’t know’ - 82%	Don’t know – 3%	< 0.001
Did you get to talk to patients yourself?	Yes – 88%	Yes – 94%	0.120
Did you feel some patients had Mental Health issues?	Yes – 73%	Yes – 90%	0.002
Did you discuss Mental Health issues with patients?	Yes – 31%	Yes – 62%	< 0.001
Was the knowledge provided in PMHD relevant to patient discussions around Mental Health on rural placement?	n.a.	Yes – 85%	–
Did any of the practical skills provided in PMHD come in useful on your Year 2 rural placement?	n.a.	Yes – 88%	–

^a^Chi-square test (2-sided) or T-test (2-sided), as appropriate

### Analysis and statistics

All data were coded numerically and entered into the computerized statistical package for social sciences, SPSS Release 20 for Windows (IBM Corp, Release 2011; http://www.spss.com). Bivariate analyses between student clinical experiences on rural placement and whether students had undertaken the Year 2 clinical placement before or after the PMHD course were assessed using two-tailed, paired T-tests and chi-squared tests, as appropriate (Table 1). Statistical tests were considered significant with *p*-values < 0.05.

The variety of responses given to the 2 open-ended questions: ‘what information provided in PMHD was relevant to the patient discussions?’, and ‘what practical skills provided in PMHD came in useful during your rural placement?’ were later grouped ‘a posteriori*’* based on the students’ factual experiences on placement, using simple content analysis (displayed in Table 2). The content analysis of the open-ended questions was conducted by the author TW, and this analysis was checked by CRB for investigator triangulation; differences were resolved through discussion between the two authors.

**Table 2:** Psychological Medicine and Human Development (PMHD) lecture content and practical skills reported by Year 2 James Cook University medical students as ‘being useful’ on their 4-week rural clinical placement (data derived from open-ended question)

Lecture content	Number of students who reported this being a useful aspect of PMHD (including % of the 97 students whom responded to this question)
1) Assessing mental illness; including MSE, mini-MSE and ‘A SAD WISH’ mnemonic	49 (51%)
2) General information on mental health (‘all was relevant’)	20 (21%)
3) Depression, loss and grief	21 (22%)
4) Interviewing mental health patients; e.g., how to approach the topic, history-taking, communication, talking to family	15 (15%)
5) Anxiety	10 (10%)
6) Lifespan development in relation to youth mental health	10 (10%)
7) Personality disorders; e.g., borderline personality disorder	8 (8%)
8) Managing mental illness; e.g., intervention strategies, care plans, medications	7 (7%)
9) Suicide prevention	5 (5%)
10) Elder health; e.g., dementia	4 (4%)
11) Psychoses; e.g., schizophrenia	4 (4%)
12) PTSD	3 (3%)
Practical skills	Number of students whom reported this being a useful aspect of PMHD (including % of the 100 students whom responded to this question)
1) How to undertake a MSE or mini-MSE; including ‘A SAD WISH’ and other mnemonics for interviewing patients with depression (appetite, sleep, affect, diurnal variation, weight, interests, suicidal thinking, hopelessness)	89 (89%)
2) Interviewing skills	20 (20%)
3) How to identify patients with a mental illness	5 (5%)
4) Suicide awareness, talking about suicide, and assessing patients with suicidal thoughts	4 (4%)

## Results

### Description

A total of 209 JCU MBBS Year 2 students participated in the study from a possible 217 (response rate = 96%). Of the 209 students, 173 (83%) sat in with a GP during a patient consultation; of these, 161 (93%) reported that the topic of the patient’s mental health was raised during a patient consultation. One-hundred and ninety students (91%) were also given the opportunity by their supervising General Practitioner (GP) to interview patients independently, with 95 (49%) discussing a patient’s mental health issue in some way.

Of the 118 students who completed their rural placement at the end of year 2014 after undertaking PMHD, 100 (85%) reported that the knowledge provided in PMHD was relevant to patient discussions around mental health, and 88% reported that practical skills learnt in PMHD were useful during their rural placement.

### Bivariate analysis

The bivariate analysis in Table 1 shows Year 2 JCU medical students who undertook their rural clinical placement at the end of 2014 after completing PMHD: felt more comfortable discussing mental health issues with patients (*p* = 0.001); were more likely to discuss mental health issues with patients (*p* < 0.001); felt better prepared to discuss mental health issues with a patient (*p* < 0.001); felt more confident in being able to detect patients with a mental health issue (*p* < 0.001); were more likely to report they believed some patients had mental health issues (*p* = 0.002); and were more likely to volunteer an opinion on the appropriateness of their supervising doctor’s response (*p* < 0.001), compared to JCU medical graduates who undertook their rural clinical placement before PMHD in mid-2014.

### Content analyses

Table 2 categorizes the PMHD lecture content and practical skills that Year 2 JCU medical students reported useful on their rural clinical placement. Overall, 97 Year 2 students (82%) reported finding at least one aspect of the lecture content of PMHD to be useful; most commonly this was around assessing mental illness (51%) and specific information on ‘depression, loss and grief’ (20%), while 21% reported ‘all was relevant’. Similarly, 100 Year 2 students (85%) found at least one practical skill to be useful, with the most useful practical skills being ‘how to undertake a Mental State Exam (MSE) or mini-MSE’ (89%), and ‘how to interview patients’ (20%).

For the open-ended question ‘why did/didn’t you feel prepared for the patient discussions around mental health?’, several student comments were taken verbatim from the surveys to illustrate why students who had undertaken PMHD didn’t always feel prepared for patient discussions around mental health, even though as a group they reported feeling significantly better prepared than students who had not undertaken PMHD. Typical comments included:

*“PHMD prepared me to handle certain patients with mental illnesses, but because it is a sensitive/daunting consultation, I felt only a little ready.”*

*“You know they have some very complex problems going on in their life and you don’t want to say anything wrong. I feel very young and inexperienced”.*

*“The PMHD subject really helped. [But] I didn’t feel quite prepared as it was my first time experiencing real life practise with the Mental State Exam.”*

*“Had a 17 year old male who had come in for gynecomastia but was very suicidal – I felt I wouldn’t have been equipped to talk to him as he was really intense”.*

*“There are a lot of things that can simply not be prepared for in theory, such as the physical interactions with patients, particularly in schizophrenia cases”.*

## Discussion

This study demonstrates JCU medical students who undertook a psychological medicine course with lecture content on the mental health issues prevalent in rural areas and practical experiences around discussing and assessing these issues with patients, reported being significantly better prepared for interacting with patients having psychological distress than students who went on their rural placement before undertaking the psychological medicine course. While it is not new that the medical students should be equipped to deal with disorders of mental health, it is new that medical students are reporting that they are benefiting from early undergraduate exposure to practical and relevant mental health knowledge and competencies.

As with other streams of the medical curriculum at JCU, there has been an attempt to build psychological medicine and associated clinical skills in an integrated systems-based fashion. PMHD now combines a relevant chronological curriculum for understanding psychological development and health across the lifespan. Students are taught how to conduct a thorough mental state examination with regard to psychiatric conditions across the life-span, and how to conduct a cognitive assessment using the mini-mental state. With this curriculum change, PMHD should now provide medical students with relevant and practical mental health knowledge and competencies for their extensive hospital and medical centre placements in Years 2, 4, 5 and 6. On these rural placements, it is almost certain the students will come into direct contact with patients experiencing psychological distress, and/or be present in the room where a doctor will model general skills in interviewing patients with psychological distress as part of the general consult. Sometimes, the medical student will even be asked to undertake a mental health assessment and interview by themselves.

Indeed, the content analyses shows 82% and 85% of the students who went on their Year 2 placement after undertaking PMHD reported at least one aspect of the lecture content and the practical sessions, respectively, came in useful when discussing mental health with patients experiencing psychological distress. In particular, students found the information on locally prevalent mental health conditions and practical experiences in the classroom around interviewing patients about these conditions and conducting depression and anxiety assessment and mental state examinations to be of most use.

However, it is not expected that a single semester subject in year two of an undergraduate medical course would fully prepare students for detecting, diagnosing and treating disorders of mental health in medical practice. Indeed, this study found many students reporting they did not feel fully prepared for discussing mental health with patients experiencing psychological distress even after undertaking PMHD. Some students found the topic too sensitive or subject material confronting (e.g., sexual abuse, refugees with PTSD), while others were concerned about saying the wrong thing to either make the situation worse for depressed or suicidal patients, or potentially turn psychotic patients violent.

Australian medical students will regularly treat patients with disorders of mental health in general practice or on hospital wards because such disorders are common in Australian populations; especially rural and remote communities. Although often confronting, medical students are able to grasp and appreciate the many aspects of mental health in different settings if provided with the appropriate relevant and practical mixture of mental health knowledge and competencies.

It is also suggested that this novel methodology – aligning formal teaching in a subject with an evaluation utilizing a proximate student placement to provide useful feedback on the curriculum content and assess the relevance of the material taught and improve the delivery of appropriate practical competencies – could be used to revise other content areas of a medical course to be more locally relevant and practically focused, and then to evaluate the success of this revision.

### Limitations

While the nature of the JCU MBBS program is not representative of all medical schools across Australia or the world, it is expected that there will be some commonality between PMHD and the teaching of psychological medicine in other medical courses. Thus, the main limitation of the study is likely to be that while the data collection method was developed and tested for validity with Year 3 medical students, it was not formally tested for reliability.

In addition, it is uncertain whether the proportion of consults for students who went on their rural placement pre-PMHD subject are comparable to those in the post-PMHD group; that is, whether the proportion of consults warranting some mental health query would have been the same. However, given the prevalence of patients presenting with mental health issues in Australian rural practice [4, 5], the proportion is likely to be similar.

Finally, while post-hoc analyses were undertaken that showed no differences in regards to gender and end-of-year academic achievement between the 2 groups of medical students who went on placement pre- versus post-PMHD course, it may be that the second group (which received the PMHD course) had better results for interacting with patients experiencing a mental health condition because of their extra 6 months of the Year 2 curriculum building their clinical skills and confidence, rather than from the PHMD course itself.

## Conclusions

Australian medical students who are provided psychological medicine information on locally prevalent mental health conditions, practical classroom experiences conducting cognitive assessments and mental state examinations for depression and anxiety, are significantly better prepared for interacting with patients in the local region experiencing psychological distress, than are students whom are not provided these experiences.
